# Japan Trial in High-Risk Individuals to Enhance Their Referral to Physicians (J-HARP)—A Nurse-Led, Community-Based Prevention Program of Lifestyle-Related Disease

**DOI:** 10.2188/jea.JE20180194

**Published:** 2020-04-05

**Authors:** Midori Noguchi, Sumi Kojima, Toshimi Sairenchi, Minako Kinuta, Miyae Yamakawa, Hitoshi Nishizawa, Mitsuyoshi Takahara, Hironori Imano, Akihiko Kitamura, Toshiko Yoshida, Ayumi Shintani, Isao Saito, Tetsuji Yokoyama, Iichiro Shimomura, Hiroyasu Iso

**Affiliations:** 1 Public Health, Department of Social Medicine, Graduate School of Medicine Osaka University, Osaka, Japan; 2 Amagasaki City Office, Hyogo, Japan; 3 Department of Public Health, Dokkyo Medical University School of Medicine, Tochigi, Japan; 4 Department of Health Sciences, Osaka University Graduate School of Medicine, Osaka, Japan; 5 Department of Metabolic Medicine, Graduate School of Medicine Osaka University, Osaka, Japan; 6 Tokyo Metropolitan Institute of Gerontology, Tokyo, Japan; 7 School of Nursing, Miyagi University, Miyagi, Japan; 8 Department of Medical Statistics, Graduate School of Medicine Osaka City University, Osaka, Japan; 9 Department of Community Health Systems Nursing, Ehime University Graduate School of Medicine, Ehime, Japan; 10 National Institute of Public Health, Saitama, Japan

**Keywords:** health checkup, health counselling, referral, physicians, risk, lifestyle-related disease, community, clustered randomized trail

## Abstract

**Background:**

It is uncertain whether health counselling after community-based health checkups for high-risk individuals of lifestyle-related disease enhances their referral to physicians.

**Methods:**

We performed a clustered randomized controlled trial of untreated high-risk individuals aged 40 to 74 years who were screened from the annual health checkup in 2014 and 2015 under the national health insurance in 43 municipalities around Japan, assigning 21 intervention and 22 usual care municipalities. The high-risk conditions were severe forms of hypertension, diabetes, dyslipidemia (for men), and proteinuria. For the intervention group, the theory-based health counselling was performed to enhance referrals to physicians, while each municipality performed its own standard counselling for the usual care group. Data on clinical visits and risk factors were collected systematically and anonymously from the databases of health insurance qualification, health insurance claims, and annual health checkups. Hypotheses are that the cumulative proportion of seeing physicians (clinical visits) is higher in the intervention than the usual care groups, and that those in the intervention group have lower cumulative incidence of composite outcomes associated with lifestyle-related diseases.

**Results:**

The numbers of subjects for the analyses were 8,977 in the intervention group and 6,733 in the usual care group. Among them, 6,758 had hypertension, 2,147 had diabetes, 2,861 had dyslipidemia, and 1,221 had proteinuria in the intervention group, with corresponding numbers of 4,833, 1,517, 2,262, and 845, respectively, in the usual care group. There were no material differences in mean levels and proportions of major cardiovascular risk factors between the two groups.

**Conclusions:**

We expect to provide scientific evidence on the effectiveness of health counselling.

## INTRODUCTION

Around 35% of deaths in Japan are from cardiovascular disease, chronic kidney disease and renal failure, and a quarter of total medical expenditure is associated with these diseases, so their prevention and control is an important issue in Japan.^1^ A system of specific health checkups and guidance for men and women aged 40 to 74 years was launched in 2008 under the national health insurance.^2^ This system screens for high-risk individuals with metabolic syndrome, high LDL-cholesterol, and cigarette smoking and helps them to reduce their risk through lifestyle changes and, if the expected risk is high enough, to refer them to physicians for treatment in the prevention of cardiovascular disease and chronic kidney disease. However, it is uncertain whether referrals to physicians are carried out effectively and sufficiently. Approximately 40% of patients with untreated severe hypertension identified through the health checkup did not see a physician after that,^3^ and over half of patients with incident cardiovascular disease had not seen a physician to seek treatment for high risk factors before the onset of cardiovascular disease.^4^

We constructed a model for the enhanced referral of high-risk individuals to physicians. The model combines the health belief model^5^ with a health counselling method developed by a municipal public health department in Amagasaki city.^5^ We tested the effectiveness of this model for high-risk individuals identified through annual health checkups who were likely to develop cardiovascular disease or renal failure. Our a prior hypotheses are that high-risk individuals in the intervention group see a physician more than those in the usual care group, and that those in the intervention group have the lower cumulative incidence of composite outcomes associated with lifestyle-related diseases.

## METHODS

### Primary outcomes

The trial has two primary outcomes: the cumulative proportion of participants’ clinical visit and the cumulative incidence of composite outcomes (ie, hospitalization from stroke, myocardial infarction, unstable angina, heart failure, chronic kidney disease/failure, and artificial dialysis, sudden cardiac death and death from cardiovascular disease, chronic kidney disease/failure, ischemic heart disease, chronic kidney failure, or artificial dialysis).

### Sample size calculation

To test the hypothesis on the outcome of cumulative proportion of participants’ clinical visit, we need only 90 high-risk individuals in each of the intervention and control groups in order to detect the difference in the proportion between 80% in the intervention and 60% in the usual care groups under the significant level (two-tailed) of 0.05 and statistical power of 0.80. To test the hypothesis on the outcome of the cumulative incidence of composite outcomes, we estimated a need for 43 municipalities of 400 participants each in order to detecta 20% lower 4-year cumulative proportion of composite outcome from the level of 6.6% in the intervention than in the usual care groups under the significance level (two-tailed) of 0.05 and statistical power of 0.80, with clustered inter-correlation of 0.001. To maintain enough statistical power, we decided to extend the follow-up from 4 years to 4.5 years.

### Study participants and randomization

This trial was designed as a two-armed randomized controlled trial in community settings. The current trial was registered at the University Hospital Medical Information Network (UMIN) Clinical Trials Registry (UMIN-CTR; https://www.umin.ac.jp/ctr/), with the unique ID UMIN000014012. We recruited participants from municipalities with over 2,000 people aged 40 to 74 years in fiscal years 2012 or 2013 who received health checkups under the national health insurance.

A total of 43 municipalities were assigned to either intervention (21 municipalities) or usual care (22 municipalities) groups via cluster randomization (eFigure 1). Among the 43 municipalities, 28 were assigned to 14 intervention and 14 usual care conditions where the intervention was performed between April 2014 and March 2016; 3 intervention and 3 usual care conditions where the intervention was done between September 2014 and March 2016; and 4 intervention and 5 control conditions where the intervention was done between April 2015 and March 2016.

In order to provide balance between the two groups, the randomization was performed within a set of two municipalities which were matched as having a closest multivariable Mahalanobis distance^6^ by a well-trained statistician at Vanderbilt University in the United States who was blinded to the names of municipalities. Municipalities were matched on characteristics, including longitude, latitude, the number of high-risk individuals, the number of persons with the national health insurance, the participation rate of specific health checkups, the proportion of educational attainment, the number of physicians per 100,000 population in each municipality, as well as the number of similar components of our trial health counselling.

There were no persons falling under exclusion criteria, such as having difficulty in receiving health counselling due to cognitive impairments, other psychiatric disorders, hearing and visual impairments, and other reasons.

High-risk individuals were assigned to the intervention or control group by municipality. Opt-out in the study was carried out through the web sites of all participating municipalities and Osaka University, providing the description of the study and the method for posting refusal through the web.

This trial was performed according to laws on personal information protection and ethical guidelines on epidemiological research, which was approved by the Osaka University Ethics Committee (No. 13237-6). The data entry of the trial was completed on Oct 27, 2016.

### High-risk individuals

The health checkups included the questionnaire, interview, physical examinations and measurements of height and weight, waist circumference, blood pressure, LDL-cholesterol, HbA1c, and blood glucose under the standardized ways prescribed by the Ministry of Health, Labour and Welfare.^7^

The interview queried smoking status for the response to non- and current smoking; drinking status for the response to never, sometime, and daily drinking; medication uses for hypertension, diabetes, and dyslipidemia; and histories of stroke, ischemic heart disease, and chronic kidney disease (and/or artificial dialysis). Height and weight were measured in light clothing.

High-risk individuals were defined as persons with at least one of the following results at a health checkup, but who had not seen physicians for the following identified conditions, drawn from clinical practice guidelines by the Japanese Society of Hypertension, the Japan Diabetes Society, Japan Atherosclerosis Society, and Japanese Society of Nephrology:

1) Grade II or more hypertension: systolic blood pressure of 160 mm Hg or more or diastolic blood pressure of 100 mm Hg or more;
2) Diabetes mellitus: glycated hemoglobin A1c of 7% or more based on the National Glycohemoglobin Standardization Criteria. If glycated hemoglobin A1c level was not measured, fasting glucose of 130 mg/dL or more. If fasting glucose level was not measured either, non-fasting glucose of 180 mg/dL or more;
3) For men, low-density lipoprotein (LDL) cholesterol of 180 mg/dL or more; and
4) Proteinuria of +2 or more in urinalysis.

### The model for enhanced referral of high-risk individuals to physicians

**Figure 1.**
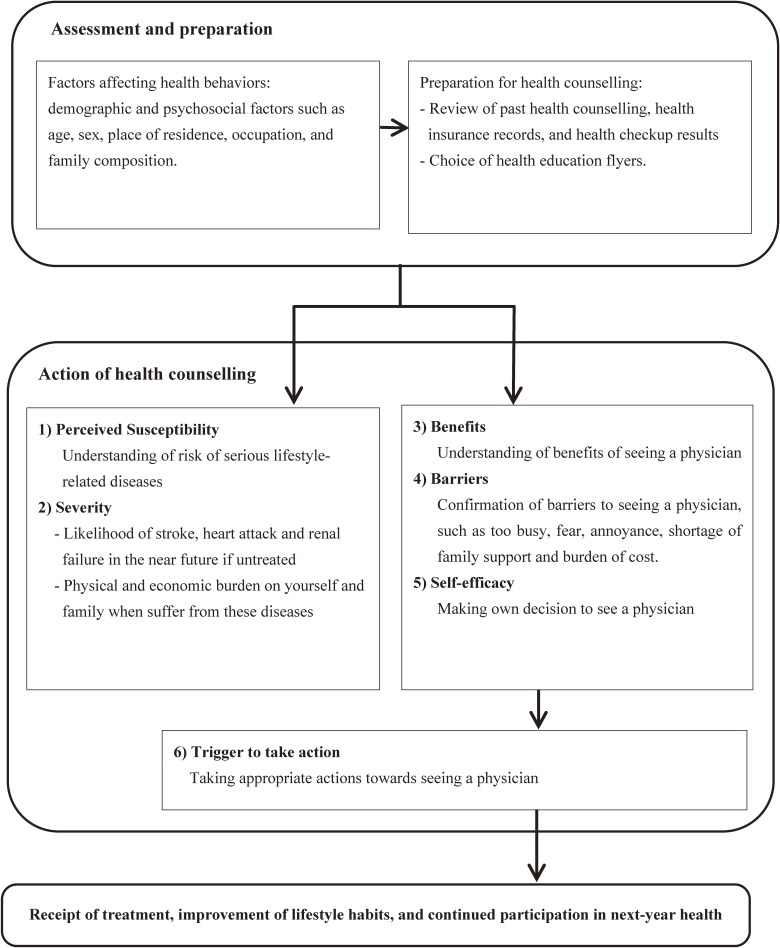
The model for enhanced referral of high-risk individuals to physicians

The model for health counselling in the intervention group was designed to provide high-risk individuals with information about what is happening in their bodies and blood vessels and about their future risk of cardiovascular disease and renal failure (Figure 1). Before counselling, a public health nurse (PHN) or a certified nutritionist or a trained nurse collected information about individual demographic and psychosocial factors, such as age, sex, place of residence, occupation, and family composition, that may affect health behaviors and prepared a plan for health counselling using the interpretation of health checkup results and health insurance claims, and then chose health education flyers.

During the counselling, the PHN explained normal physiology, and how disorders in the body and vessels result from high blood pressure, hyperglycemia, and hyperlipidemia (perceived susceptibility). The PHN also provided information about how blood vessels in brain, heart, and kidneys will be damaged and what serious health problems, including stroke, heart attack, and renal failure, are likely to happen in the near future if left untreated (severity). It is also stressed that these diseases would harm his or her life physically and economically (severity). The PHN then provides information to help participants understand the benefits of seeing a physician (benefits), and asks about any barriers to prevent this action, such as being too busy, fear, annoyance, shortage of family support, and cost (barriers). Accordingly, high-risk individuals are expected to make their own decisions (self-efficacy) and take the appropriate action, such as seeing a physician to seek further counselling and treatment (trigger to action). Then, participants are expected to receive treatment, improve lifestyle habits, and continue to participate in next-year health checkups.

### Supplementary tools for the health counselling

**Figure 2.**
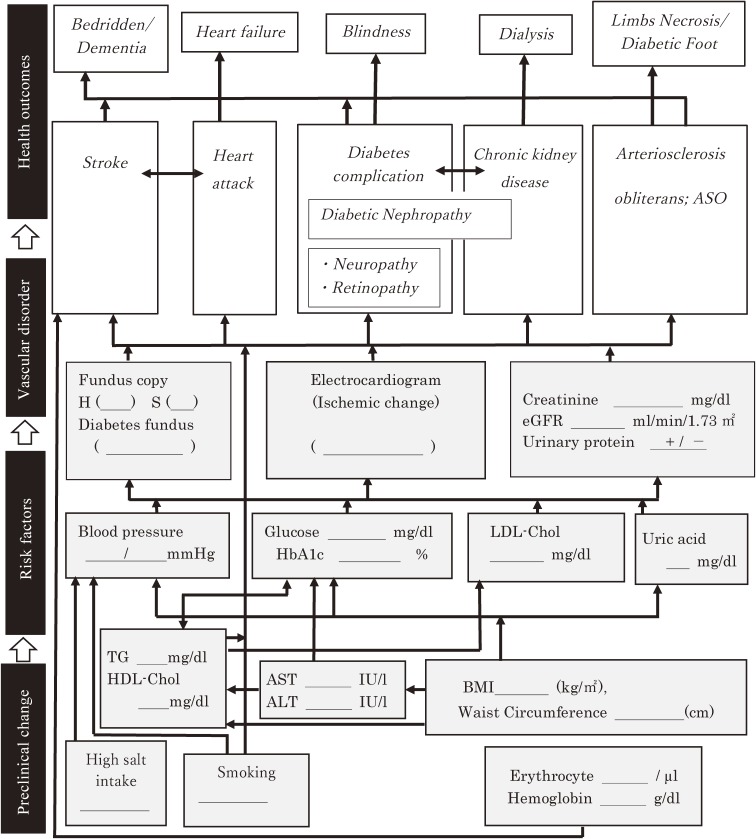
Upgraded ‘Where am I?’ chart (‘Flow of disease progression’ chart). The underline parts will be filled in by the data of each subject.

The PHN hands the participant two result forms of the health checkup: 1) a sheet of results over the past 5 years, and 2) as shown in Figure 2, a flow chart of risk behaviors (high salt intake and smoking), and metabolic risk factors (body mass index, waist circumference, triglycerides, HDL-cholesterol, blood pressure, hemoglobin A1c/glucose, LDL-cholesterol, and uric acid), preclinical vascular disorders (hypertensive and diabetic funduscopic findings, resting electrocardiogram findings, urinary protein serum, and, if available, creatinine and estimated glomerular filtration rate), diseases (stroke; heart attack; diabetes complications, such as nephropathy, neuropathy, retinopathy, and chronic kidney disease; and peripheral vascular disease, such as arteriosclerosis obliterans), and end-stage conditions, such as heart failure, blindness, dialysis, bedridden condition, dementia, and necrosis of extremities (Figure 2).

This progression chart was developed from the “Where am I?” chart, created by the public health department of Amagasaki city to improve the control of metabolic syndrome for the prevention of cardiovascular disease.^6^ We modified it so that it can also be applied to non-obese high-risk individuals. We also developed 34 types of flyers as subsidiary materials for the intervention (http://www.pbhel.med.osaka-u.ac.jp/common/images/pdf/themes/jharp/hokenshidou.pdf). These flyers were linked to the study model of health counselling, and they were chosen and used to explain more about particular risks. They included, for example, “What is the cause of injured blood vessels?”, and “What stage of blood vessel injury do your results correspond to?”. The shape of the blood vessel and its damage were shown in color, so that participants would understand their upcoming risk of developing cardiovascular disease and kidney failure requiring medical treatment.

### Statistical analysis

To test differences in the baseline characteristics of major risk factors between the intervention and usual care groups, we used *t*-test for mean values and chi-square test for proportions. All statistical testing was performed using SAS 9.3 (SAS Institute Inc., Cary, NC, USA), and tests were two-tailed, with *P* values below 0.05 considered to be statistically significant. Additional description of the methods is shown in eAppendix 1.

## RESULTS

There were 10,519 high-risk individuals in the intervention group and 8,353 in the usual care group (eFigure 1). We excluded 1,517 (12.6%) in the intervention and 1,127 (12.5%) in the usual care groups who had already seen a physician or received medication for any of the high-risk factors, such as hypertension, diabetes, dyslipidemia, and chronic kidney disease, and anyone aged under 40 or over 74 years. There was no significant difference in the proportion of person who has already seen a physician between the two groups (*P* = 0.85). This left 8,977 in the intervention group and 6,733 in the usual care group for the analyses.

As shown in Table 1, mean age at baseline was 63–64 years old, and the proportion of men were 67–68% in intervention and usual care groups, respectively. The proportions of overweight, grade II or more hypertension, hyperglycemia, and high LDL-cholesterol and proteinuria were 33–34%, 57–55%, 19%, 23–25%, and 10–9%, respectively. The proportions of current smokers, current drinkers, and overweight were 19–21%, 55–52%, and 33–34% respectively. There was no difference in means and proportions of variables for clustered randomization between the intervention and usual care municipalities.

**Table 1.** Means and proportions of baseline characteristics in the intervention and usual care groups and of variables for randomization

	Intervention	Usual care	*P*-value
Number of participants, *n*	8,799	6,733	
Age, years	63.3 (8.48)	63.8 (8.07)	<0.0001
Men, %	66.4	67.6	0.04
Body mass index, kg/m^2^	23.9 (3.53)	23.9 (3.49)	0.67
Waist circumference, cm	85.1 (9.15)	85.4 (9.19)	0.03
Overweight (body mass index ≥25 kg/m^2^), %	33.4	33.6	0.33
Grade II or more hypertension,^a^ %	57.4	55.4	0.009
Hyperglycemia (HbA1c ≥7.0%),^b^ %	19.1	18.8	0.59
High-LDL cholesterol among men,^c^ %	23.2	25.1	0.003
Proteinuria 2+ or more, %	10.3	9.4	0.14
Current smokers, %	19.3	20.9	0.04
Current drinkers, %	54.5	52.4	0.51
Variables for clustered			
Number of municipalities, *n*	21	22	
Number of population, *n*	187,249 (202,492)	174,266 (168,463)	0.89
Number of insured persons, *n*	490,423 (53,681)	44,909 (44,498)	0.72
Number of health checkup participants, *n*	4,781.3 (2,345.9)	4,827.8 (2,939.5)	0.86
Participation rate of health checkups, %	15.05 (8.58)	16.14 (10.21)	0.95
Number of high-risk individuals, *n*	307.3 (174.1)	309.8 (182.5)	0.95
Longitude	135.5 (3.55)	136.3 (3.83)	0.47
Latitude	34.9 (1.96)	35.43 (1.98)	0.50
Number of similar components,^d^ *n* (%)			
0	1 (4.8)	3 (13.6)	0.64
1	7 (33.3)	5 (22.7)	
2	7 (33.3)	4 (18.2)	
3	6 (28.6)	10 (45.5)	
Number of physicians per 100,000, *n*	200 (77)	193 (77)	0.36
Education >12 years, %	22.5	20.8	0.54

Values are reported as mean (standard deviation), unless otherwise noted.

^a^Systolic blood pressure ≥160 mm Hg and/or dyastolic blood pressure ≥100 mm Hg.

^b^HbA1c ≥7.0%. If HbA1c are missing, fasting blood glucose ≥7.2 mmol/L (130 mg/dL). If fasting glucose levels are also missing, casual glucose levels ≥10 mmol/L (180 mg/dL).

^c^Serum LDL cholesterol among men ≥4.7 mmol/L (180 mg/dL).

^d^Similar components were 1) confirmation of the clinical visits through health insurance claims, 2) home visits for the initial health counselling, 3) use of a progress chart of vascular damage, 4) use of the present and past 5-year results of health checkups, and 5) planned and continuous health counselling by using health counselling record.

## DISCUSSION

This cluster randomized controlled trial is the first to investigate the impact of health counselling using a model to enhance referral of high-risk individuals to physicians in the prevention of cardiovascular disease and renal failure. Previous studies have examined the effects of reducing cardiovascular risk factors through health counselling,^8^ but none of them has investigated changes in the proportions of high-risk individuals seeing physicians for prevention of lifestyle-related disease.

The standardization of health counselling based on theory, the monitoring of implementation, the systematic data collection under the cluster randomization, and the large number of participants will enabled us to test our hypotheses on the acceleration of clinical visits for high-risk individuals and the lowering of the cumulative incidence of composite outcomes associated with lifestyle-related diseases.

This trial is expected to provide scientific evidence on the effectiveness of health counselling methods and tools on the referral to physicians in the prevention of severe forms of lifestyle-related disease.

## APPENDIX A. SUPPLEMENTARY DATA

eAppendix 1. Supplement for the methods

eFigure 1. Flow chart of municipalities and participants
