# Supplementary material for: Japan Trial in High-Risk Individuals to Enhance Their Referral to Physicians (J-HARP)—A Nurse-Led, Community-Based Prevention Program of Lifestyle-Related Disease
Source: J Epidemiol. 2020 Apr 5;30(4):194–9. doi: 10.2188/jea.JE20180194 (PMC7064550; doi:10.2188/jea.JE20180194)
Supplement: Supplementary file 1 [file je-30-194-s001.pdf]

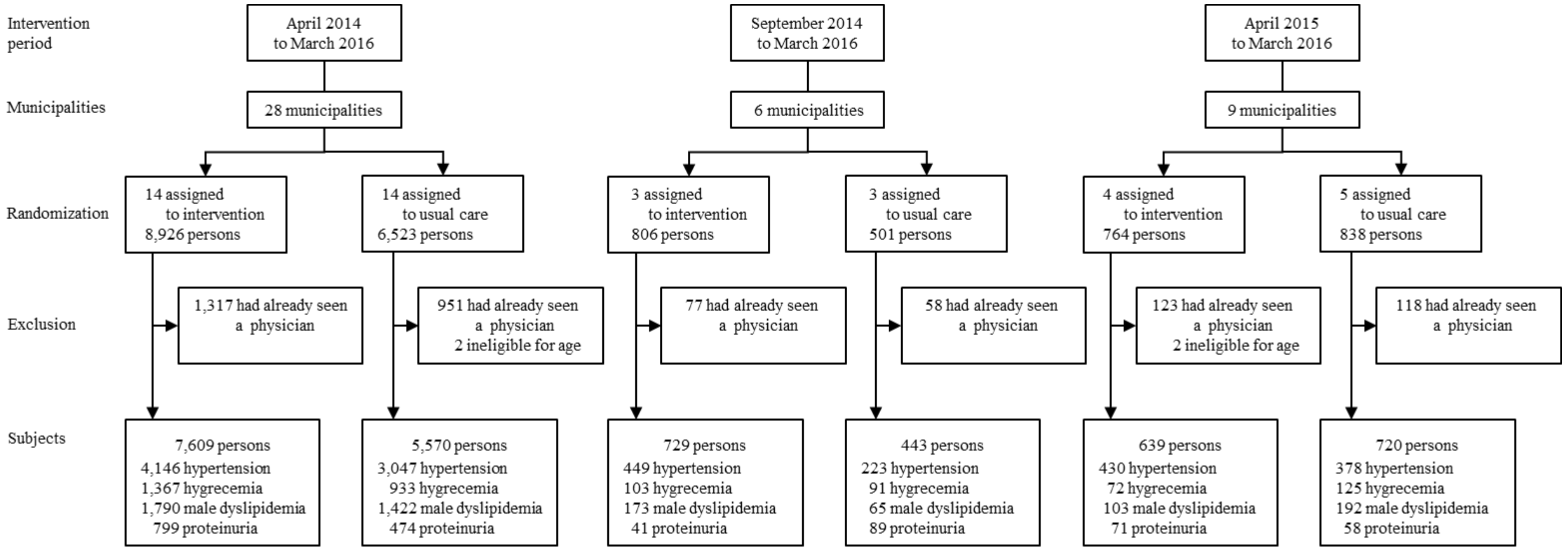

eFigure 1. Flow chart of municipalities and participants

eAppendix 1. Supplement for the methods

Standardization of the health counselling

In order to carry out the intervention at an accepted level, we provided 2-to-3-day seminars in the major cities of Tokyo, Osaka, Fukuoka, and Kagoshima on knowledge, skills, and techniques of the health counselling. The seminars were held three times a year, with e-learning available for non-attendants. We also held on-site case-study meetings by the intervention supporting team.

Monitoring for the health counselling

To confirm whether the health counselling was conducted in the standardized way, the monitoring team looked into 50 or 100 samples (depending on the size of participants) of health counselling records from each intervention municipality and reported the monitoring results to the intervention support team. The intervention support team made feedback to corresponding municipalities to enhance the quality of health counselling.

Counselling time plan

During the first year from the initial health checkup, the counselling was planned to conduct three times: 1 to 3 months, 4 to 6 months, and 7 to 9 months from the health checkup. The number of counselling in the second year varied according to health checkup results. If the result of high-risk status were improved regardless of starting medication, the counselling was conducted once or two times: 1 to 3 months and/or 7 to 9 months from the health checkup, and otherwise the same time schedule as the first year. The mode of initial health counselling was primarily home-visit counselling, secondarily face-to-face counselling at municipal office or public health center, and if these modes did not reach to the participants, then telephone counselling.

Secondary outcomes

The secondary outcomes are the proportion receiving medical treatment, the proportion of participation in the next-year health checkup, changes in mean levels of risk factors,

and the proportion of high-risk factors from the results of health checkup.

Data collection and management

Each municipality had the data of insurance qualification and collected the data of medical checkups from local bodies of health examination and the national insurance claims from the prefectural national health insurance organization. The trained staff at each municipality office used a distributed personal computer with a hard disk of tailored software in order to link those three data sets by the personal identifier and to create the corresponding table for the municipal and research identifiers. Then, the staff deleted the municipal office identifier, name, and day of birth from the datasets (but held the research identifier and birth year and month), and then restricted the data for the subjects with high risk factors (hypertension, diabetes, high male LDL-cholesterol and proteinuria) at baseline health checkups. The data were transmitted to the Data Center at National Center via registered mail. For the intervention municipalities, the health counsellors' information, the health counselling records with the research identifier were also transmitted to the Data Center via registered mail and were keyed into the digital dataset. The Data Center checked and inquired the municipalities if necessary to build the database. Using the database, the persons who reported to have medication use for the corresponding risk factors at the baseline health checkups, and/or who had the health insurance claims on hypertension, diabetes, dyslipidemia, proteinuria or chronic kidney disease, 3 months before the initial health checkups were excluded to identify the final subjects for the trial.
